# Risk Factors for Survival following Open Surgical Repair of Ruptured Abdominal Aortic Aneurysms: A 13-Year Experience

**Published:** 2015-07-03

**Authors:** Anil Ozen, Muhammet Onur Hanedan, Çetin Murat Songur, Emre Boysan, Ertekin Utku Unal, Serkan Mola, Halil Ibrahim Erkengel, Emre Kubat, Zafer Iscan, Ufuk Tutun, Ahmet Sarıtas, Cemal Levent Birincioglu

**Affiliations:** Türkiye Yüksek Ihtisas Hospital, Cardiovascular Surgery Department, Ankara, Turkey.

**Keywords:** *Aortic aneurysm*, *abdominal*, *Cardiac surgical procedures *, *Mortality*

## Abstract

**Background: **Surgical treatment of a ruptured abdominal aorta aneurysm (RAAA) continues to present a significant challenge to surgeons. There are some patient factors such as age and gender that cannot be changed, and comorbid conditions can be optimized but not eliminated. The purpose of this study was to identify the risk factors affecting high mortality after the surgical repair of an RAAA.

**Methods:** Data on 121 patients who underwent surgical repair for RAAAs between January 1997 and June 2011 in our institution were collected retrospectively. All the patients had been diagnosed by computed tomography (CT) scans, and intraoperative extra-luminal blood was visualized intraoperatively. Variables studied comprised demographic data; preoperative, operative, and postoperative data; and the causes of mortality. Multivariate regression analysis was used to determine the predictors of mortality.

**Results:** One hundred eight (89.2%) patients were male and 13 (10.7%) were female at an average age of 68.9 ± 10.5 years. Totally, 121 patients underwent surgery for RAAAs. Fifty-four patients had aortic tube grafts, 32 aortobiiliac grafts, 20 aortobifemoral grafts, 1 aortoiliac graft, and 1 aortofemoral graft for the replacement of the RAAAs. Seven patients had only surgical exploration. Operative mortality was 41.3% (50 patients). The factors associated with mortality were preoperative shock, free blood, positive inotropic agent, hematocrit value, and need for blood and plasma. In the multivariate analysis, preoperative shock and positive inotropic agents were found to be significant as the predictors of death (OR: 19.8, 95%CI: 3.2-122.8 and OR: 8.6, 95% CI: 2.9-26.3, respectively).

**Conclusion:** This study revealed that the preoperative clinical findings affected the mortality associated with RAAAs.

## Introduction

The surgical treatment of a ruptured abdominal aortic aneurysm (RAAA) continues to present a significant challenge to surgeons. The incidence of death following surgery for RAAAs remains high despite improvements in perioperative management. The perioperative mortality rate for RAAAs has remained largely unchanged at 23% to 69%.^1^

Survival after the repair of RAAAs depends on a number of patient-related factors and the management of the patient within the healthcare system.^2^^, ^^3^ There are certain patient-related factors such as age and gender that cannot be changed, and comorbid conditions can be optimized but not eliminated. The accuracy of diagnosis, time interval between the initiation of symptoms and surgery, and skill of the surgical team are among the factors related to the healthcare system. In view of the persistently high operative mortality rate, it would be advantageous to identify the preoperative and postoperative factors to predict the outcome.^4^ The purpose of this study was to identify the risk factors affecting high mortality after the surgical repair of RAAAs.

## Methods

This study was approved by the Research Committee of Turkiye Yuksek Ihtisas Training and Research Hospital, Ankara, Turkey. Informed consent was obtained from the patients or from the relatives of the patients considered incompetent to give informed consent. Data on 121 patients who underwent surgical repair for RAAAs between January 1997 and June 2011 in our institution were collected retrospectively. All the patients had been diagnosed by computed tomography (CT) scans, and intraoperative extra-luminal blood was visualized intraoperatively. Surgical exposure was gained through a midline incision. Rapid control of the RAAA was occasionally achieved proximally by manual compression. The infrarenal aorta was then cross-clamped with vascular forceps, and the clamps were then placed on the iliac arteries. All the patients were treated with aortic tube grafts or aortic bifurcation grafts, either to the iliac or to the femoral arteries. 

Surgical intervention was generally not undertaken if the patient declined the operation, had a known serious comorbidity such as advanced malignancy, or was otherwise in unsuitable conditions. These conditions included refractory loss of consciousness, cardiac arrest, severe dementia, and poor functional states.

In our study, preoperative shock was defined as persistent hypotension and the need for inotropic support despite adequate fluid resuscitation. 

Operative mortality was defined as death occurring within 30 days of surgery, whether or not the patient had been discharged from the hospital. Deaths that occurred after 30 days postoperatively without discharge were defined as in-hospital deaths. The patients were divided into survival and mortality groups. 

The preoperative factors included age, gender, creatinine and hemoglobin levels on admission, size of the aneurysm, need for inotropic support, and comorbid conditions. The intraoperative factors consisted of blood loss and operation time. The postoperative factors comprised the use of inotropic support, length of mechanical ventilatory support, length of intensive care unit (ICU) stay, respiratory failure, renal failure, neurologic complications (cerebrovascular accident or spinal cord ischemia), bowel ischemia, and lower-limb ischemia.

For the statistical analyses, the statistical software SPSS version 16.0 for Windows (SPSS Inc., Chicago, IL) was used. The results were reported as mean ± standard (SD) for the quantitative variables and percentages for the categorical variables, and the groups were compared using the Student t-test for the continuous variables and the chi-square test (or the Fisher exact test, if required) for the categorical variables. The multivariate regression analysis was used to determine the predictors of mortality. The continuous variables were normally distributed according to the Kolmogorov-Smirnov compatibility with normal distribution test.  A p value < 0.05 was considered statistically significant.

## Results

Totally, 121 patients underwent surgery for RAAAs. Fifty-four patients had aortic tube grafts, 32 aortobiiliac grafts, 20 aortobifemoral grafts, 1 aortoiliac graft, and 1 aortofemoral graft for the replacement of the RAAAs. Seven patients had only surgical exploration.

The study population comprised 108 (89.2%) males and 13 (10.7%) females at an average age of 68.9 ± 10.5 years. The patients’ demographic and preoperative data are listed in Table 1. The mean operation time was 225.2 ± 89.4 minutes. The mean ICU stay was 8.2 ± 17.3 days (0-120), and the mean duration of postoperative mechanical ventilation was 3 days (0-30 days). The mean ward stay was 10.1 ± 12.3 days (1-210 days). The operative and postoperative factors are listed in Table 2.

Operative mortality was 41.3% (50 patients). Of these 50 patients, 14 (11.5%) died in the operating room, 3 (2.4%) died within the first 24 hours in the ICU, and 33 (27.2%) died because of complications. The causes of mortality for all the cases are shown in Table 3.

The major complications after surgery included respiratory failure in 18 (14.9%) patients, renal insufficiency in 14 (11.5%), lower-limb ischemia in 11 (9.1%), bowel ischemia in 5 (4.1%) and bleeding that required reoperation in 3 (2.4%). The withdrawal of nephrotoxic drugs and renal replacement therapy was applied for the patients with renal failure following nephrology consultation. Eight patients underwent hemodialysis. The lower-limb ischemia in 4 patients resolved after heparin and low-molecular-weight heparin administration. Seven patients underwent reoperation; of these patients, 6 required femoral embolectomy and 1 required femoropopliteal bypass. The patients with bowel ischemia were consulted by the general surgery department. They all underwent surgical laparotomy, followed by bowel resection. However, all died following bowel resection.

Factors associated with mortality were preoperative shock, free blood, positive inotropic agent, hematocrit value, and blood and plasma transfusion. The mean preoperative hematocrit level was 35.7 ± 7.8% for the survival group and 30.6 ± 7.6% for the mortality group (p value = 0.001). The mean preoperative fresh frozen plasma requirement was 15.6 ± 15.4 units in the mortality group, whilst it was 4.3 ± 4.4 units in the survival group (p value < 0. 001). Similarly, the mean preoperative erythrocyte suspension requirement was 8. 7 ± 7.5 units in the mortality group, whilst it was 2. 4 ± 3.1 units in the survival group (p value < 0.001). The multivariate analysis revealed preoperative shock and positive inotropic support as the significant predictors of death (Table 4). 

**Table 1:** Demographic and preoperative characteristics*

	Without Mortality (n=71)	With Mortality (n=50)	P Value
Gender			0.825
Male	63 (88.7)	45 (90.0)	
Female	8 (11.3)	5 (10.0)	
Age (y)	67.7±10.6	70.7±10.2	0.122
HTN	69 (97.2)	49 (98.0)	0.985
DM	16 (22.5)	15 (30.0)	0.336
Smoking	38 (53.5)	23 (46.0)	0.442
COPD	16 (22.5)	9 (18.0)	0.513
CAD	21 (29.7)	11 (22.0)	0.301
PAD	19 (26.8)	12 (24.0)	0.715
Chronic Renal Failure	10 (14.1)	8 (16.0)	0.823
Preoperative shock	4 (5.6)	31 (62.0)	< 0.001
Positive inotropic support	18 (25.4)	34 (68.0)	< 0.001
Hematocrit (%)	35.7±7.8	30.6±7.6	< 0.001
Urea (mg/dl)	57.2±30.1	64.5±35.9	0.236
Creatinine (mg/dl)	1.2±0.7	1.45±0.7	0.341

HTN, Hypertension; DM, Diabetes mellitus; COPD, Chronic obstructive pulmonary disease; CAD, Coronary artery disease; PAD, Peripheral artery disease

* Data are presented as mean±SD or n (%).

**Table 2:** Operative and postoperative characteristics*

	Without Mortality (n=71)	With Mortality (n=50)	P Value
Operation time (min)	226.2±79.1	223.5±105.4	0.816
X-clamp time (min)	65.2±40.5	66.6±50.6	0.828
Free blood in abdomen	1 (1.4)	17 (34.0)	< 0.001
Erythrocyte suspension (min)	2.4±3.1	8.7±7.5	< 0.001
Fresh frozen plasma (min)	4.3±4.4	15.6±15.4	< 0.001
Ventilation time (hours)	22.7±34.3	173.4±202.3	< 0.001
ICU stay duration (day)	5.3±15.0	14.9±20.3	0.020
Ward Stay duration (day)	10.2±12.4	4.5±3.5	0.514

ICU, Intensive care unit

* Data are presented as mean±SD or n (%).

**Table 3:** Causes of mortality for all the cases (n=50)*

Bleeding	13 (26.0)
Heart failure	4 (8.0)
Respiratory failure	10 (20.0)
Multiple failure	12 (24.0)
Mesenteric ischemia	5 (10.0)
Septic complication	6 (12.0)

* Data are presented as n (%).

**Table 4:** Predictors of mortality following multivariate analysis

	P Value	Odds Ratio	%95 CI
Preoperative shock	< 0.001	19.8	3.204-122.815
Positive inotropic support	< 0.001	8.6	2.863-26.269
Free blood in abdomen	0.225	4.6	0.363-58.691
Hematocrit	0.317	1.0	0.960-1.235

## Discussion

The management of RAAAs continues to challenge vascular surgeons. The mortality rate of RAAAs remains remarkably high despite several advances in diagnostic imaging and postoperative critical care.^5^ Our overall mortality rate of 41.3% is consistent with other reports.^3^^, ^^5^^, ^^6^

Gender, age, preoperative creatinine level, operation time, and cross-clamping time are among the factors that did not differ between our survival and mortality groups. However, preoperative hemoglobin levels and need for inotropic support preoperatively were related with mortality in our series, similar to a study performed by Anain et al.^7^ Preoperative shock is the most common factor affecting survival according to previous studies. Schermerhorn et al.^8^ reported that preoperative shock was the most important factor affecting survival for both open repair and endovascular RAAA repair (EVAR). Johansen et al.^9^ found that age > 80 years, low hematocrit levels, and preoperative cardiac arrest were associated with serious mortality. Preoperative low hemoglobin levels reflected the volume of blood loss and was associated with increased mortality in our study, which chimes in with some other studies.^9^^, ^^10^ A low hemoglobin level means aggressive fluid resuscitation and blood transfusion requirement. A significant side effect of overly aggressive fluid resuscitation includes increased blood loss and dilutional and hypothermic coagulopathy.^11^ Preoperative evaluation time should be as short as possible in order to achieve aortic control as soon as possible to prevent excessive fluid replacement and blood transfusion requirements. Robert et al.^12^ suggested that a systolic blood pressure of 80-100 mmHg until aortic clamping is enough. In addition to this, Crawford^13^ stated that no significant blood volume resuscitation should be made until the time of surgery if the patient can maintain a systolic blood pressure of 50-60 mmHg.

Bowel ischemia is a fatal complication of RAAAs. Intra-abdominal hypertension is an important factor in the development of bowel ischemia. Elevated intra-abdominal pressure compresses the vena cava, liver, bowel, and kidney and worsens end-organ perfusion.^14^ Renal failure, another common fatal complication of RAAAs, is associated with substantial mortality.^15^ Respiratory failure is one of the important causes of death after open repair.^16^ In our study, we encountered respiratory failure in 18 (14.8%) patients, renal insufficiency in 14 (11.5%), lower-limb ischemia in 11 (9.1%), and bowel ischemia in 5 (4.1%). Deaths further increase when two or three complications coexist. 

The therapeutic strategies for RAAA operation have changed over the years, with relatively poor outcomes associated with open repair having driven the use of EVAR for RAAA treatment. Moreover, some surgeons have reported the advantages of EVAR for RAAAs.^17^^-^^21^ Indeed, EVAR has become more popular, and many centers perform EVAR as the first line of treatment for RAAAs in many cases.^22^ Nevertheless, the IMPROVE study did not reveal a significant reduction in 30-day mortality or cost for endovascular repair.^23^ Our study was not designed for comparing the results of open abdominal repair with EVAR. 

***Limitations***

There were several limitations to the present study. This study was not a prospective controlled randomized study. There were many other factors affecting the outcomes of the patients other than the ones which we investigated. Dueck et al.^20^ stated that the time of onset of symptoms to admission to the emergency department, surgeon volume and training, hospital type and volume, and time of day of operation were also effective on mortality. A more detailed study of these issues is needed.

## Conclusion

This study showed that preoperative clinical findings affected the mortality associated with RAAAs in our patients. Even though the mortality rates for this condition remain high, careful consideration and management of preoperative and postoperative factors may help further reduce mortality and morbidity rates.

## Notes:

***This paper should be cited as: ***Ozen A, Hanedan MO, Songur ÇM, Boysan E, Unal EU, Mola S, Erkengel HI, Kubat E, Iscan Z, Tutun U, Sarıtas A, Birincioglu CL. Risk Factors for Survival Following Open Surgical Repair of Ruptured Abdominal Aortic Aneurysms: A 13-Year Experience. J Teh Univ Heart Ctr 2015;10(3):117-121.

## References

[1] Ernst CB (1993). Abdominal aortic aneurysm. N Engl J Med.

[2] Heikkinen M, Salenius JP, Auvinen O (2002). Ruptured abdominal aortic aneurysm in a well-defined geographic area. J Vasc Surg.

[3] Bown MJ, Sutton AJ, Bell PR, Sayers RD (2002). A meta-analysis of 50 years of ruptured abdominal aortic aneurysm repair. Br J Surg.

[4] Alric P, Ryckwaert F, Picot MC, Branchereau P, Colson P, Mary H, Marty-Ané C (2003). Ruptured aneurysm of the infrarenal abdominal aorta: impact of age and postoperative complications on mortality. Ann Vasc Surg.

[5] Scarcello E, Ferrari M, Rossi G, Berchiolli R, Adami D, Romagnani F, Mosca F (2010). A new preoperative predictor of outcome in ruptured abdominal aortic aneurysms: the time before shock (TBS). Ann Vasc Surg.

[6] Noel AA, Gloviczki P, Cherry KJ, Jr, Bower TC, Panneton JM, Mozes GI, Harmsen WS, Jenkins GD, Hallett JW, Jr (2001). Ruptured abdominal aortic aneurysms: the excessive mortality rate of conventional repair. J Vasc Surg.

[7] Anain PM, Anain JM, Sr, Tiso M, Nader ND, Dosluoglu HH (2007). Early and mid-term results of ruptured abdominal aortic aneurysms in the endovascular era in a community hospital. J Vasc Surg.

[8] Schermerhorn ML, Bensley RP, Giles KA, Hurks R, Oʼmalley AJ, Cotterill P, Chaikof E, Landon BE (2012). Changes in abdominal aortic aneurysm rupture and short-term mortality, 1995-2008: a retrospective observational study. Ann Surg.

[9] Johansen K, Kohler TR, Nicholls SC, Zierler RE, Clowes AW, Kazmers A (1991). Ruptured abdominal aortic aneurysm: the Harborview experience. J Vasc Surg.

[10] Nakayama A, Morita H, Miyata T, Hoshina K, Nagayama M, Takanashi S, Sumiyoshi T, Komuro I, Nagai R (2014). Predictors of mortality after emergency or elective repair of abdominal aortic aneurysm in a Japanese population. Heart Vessels.

[11] Bickell WH, Wall MJ, Jr, Pepe PE, Martin RR, Ginger VF, Allen MK, Mattox KL (1994). Immediate versus delayed fluid resuscitation for hypotensive patients with penetrating torso injuries. N Engl J Med.

[12] Roberts K, Revell M, Youssef H, Bradbury AW, Adam DJ (2006). Hypotensive resuscitation in patients with ruptured abdominal aortic aneurysm. Eur J Vasc Endovasc Surg.

[13] Crawford ES (1991). Ruptured abdominal aort aneurysm. J Vasc Surg.

[14] Kunishige H, Ishibashi Y, Kawasaki M, Morimoto K, Inoue N (2013). Risk factors affecting survival after surgical repair of ruptured abdominal aortic aneurysm. Ann Vasc Dis.

[15] Harris LM, Faggioli GL, Fiedler R, Curl GR, Ricotta JJ (1991). Ruptured abdominal aortic aneurysms: factors affecting mortality rates. J Vasc Surg.

[16] Tilney NL, Bailey GL, Morgan AP (1973). Sequential system failure after rupture of abdominal aortic aneurysms: an unsolved problem in postoperative care. Ann Surg.

[17] Ohki T, Veith FJ (2000). Endovascular grafts and other image-guided catheter-based adjuncts to improve the treatment of ruptured aortoiliac aneurysms. Ann Surg.

[18] Mehta M, Taggert J, Darling RC, 3rd, Chang BB, Kreienberg PB, Paty PS, Roddy SP, Sternbach Y, Ozsvath KJ, Shah DM (2006). Establishing a protocol for endovascular treatment of ruptured abdominal aortic aneurysms: outcomes of a prospective analysis. J Vasc Surg.

[19] Ricotta JJ, 2nd, Malgor RD, Oderich GS (2010). Ruptured endovascular abdominal aortic aneurysm repair: part II. Ann Vasc Surg.

[20] Zeng QL, Yang GH, Ni L, Lai ZC (2014). Comparison of endovascular aortic repair and open surgical repair for ruptured abdominal aortic aneurysm. Acta Acad Med Sin.

[21] Gullu AU, Burnaz T, E Okten, S Senay, Arıturk C, Toraman F, Kocyigit M, Yaylaci S, Karabulut E, Alhan C (2014). Emergentendovascularrepairof ruptured abdominal and thoracic aortic aneurysms in a single center: midtermoutcomes. Chirurgia.

[22] Dueck AD, Kucey DS, Johnston KW, Alter D, Laupacis A (2004). Survival after ruptured abdominal aortic aneurysm: effect of patient, surgeon, and hospital factors. J Vasc Surg.

[23] IMPROVE Trial Investigators, Powell JT, Sweeting MJ, Thompson MM, Ashleigh R, Bell R, Gomes M, Greenhalgh RM, Grieve R, Heatley F, Hinchliffe RJ, Thompson SG, Ulug P (2014). Endovascular or open repair strategy for ruptured abdominal aortic aneurysm: 30 day outcomes from IMPROVE randomised trial. BMJ.

